# Quantifying uncertainty of molecular mismatch introduced by mislabeled ancestry using haplotype-based HLA genotype imputation

**DOI:** 10.3389/fgene.2024.1444554

**Published:** 2024-09-25

**Authors:** Benedict M. Matern, Eric Spierings, Selle Bandstra, Abeer Madbouly, Stefan Schaub, Eric T. Weimer, Matthias Niemann

**Affiliations:** ^1^ PIRCHE AG, Berlin, Germany; ^2^ Center for Translational Immunology and Central Diagnostics Laboratory, University Medical Center, Utrecht, Netherlands; ^3^ Center for International Blood and Marrow Transplant Research, Minneapolis, MN, United States; ^4^ Clinic for Transplantation Immunology and Nephrology, University Hospital Basel, Basel, Switzerland; ^5^ Transplantation Immunology, Department of Biomedicine, University of Basel, Basel, Switzerland; ^6^ HLA-Diagnostics and Immunogenetics, Department of Laboratory Medicine, University Hospital Basel, Basel, Switzerland; ^7^ Department of Pathology and Laboratory Medicine, University of North Carolina at Chapel Hill School of Medicine, Chapel Hill, NC, United States; ^8^ Molecular Immunology Laboratory, McLendon Clinical Laboratories, UNC Hospitals, Chapel Hill, NC, United States

**Keywords:** imputation, HLA, epitope, PIRCHE, ancestry, bioinformatics, haplotype

## Abstract

**Introduction:**

Modern histocompatibility algorithms depend on the comparison and analysis of high-resolution HLA protein sequences and structures, especially when considering epitope-based algorithms, which aim to model the interactions involved in antibody or T cell binding. HLA genotype imputation can be performed in the cases where only low/intermediate-resolution HLA genotype is available or if specific loci are missing, and by providing an individuals’ race/ethnicity/ancestry information, imputation results can be more accurate. This study assesses the effect of imputing high-resolution genotypes on molecular mismatch scores under a variety of ancestry assumptions.

**Methods:**

We compared molecular matching scores from “ground-truth” high-resolution genotypes against scores from genotypes which are imputed from low-resolution genotypes. Analysis was focused on a simulated patient-donor dataset and confirmed using two real-world datasets, and deviations were aggregated based on various ancestry assumptions.

**Results:**

We observed that using multiple imputation generally results in lower error in molecular matching scores compared to single imputation, and that using the correct ancestry assumptions can reduce error introduced during imputation.

**Discussion:**

We conclude that for epitope analysis, imputation is a valuable and low-risk strategy, as long as care is taken regarding epitope analysis context, ancestry assumptions, and (multiple) imputation strategy.

## Introduction

Many modern histocompatibility algorithms are dependent on analysis of complete HLA protein structures (Senev et al., 2020). This is especially true when considering epitope-based algorithms, as they aim to understand the mechanics of specific physico-chemical interactions involved in, e.g., antibody or T cell-receptor binding (Nielsen et al., 2007; Kosmoliaptsis et al., 2011). An incomplete or incorrect categorization of the HLA structure could lead to inaccurate epitope predictions.

High-resolution HLA genotype imputation can be performed in those cases where only low/intermediate-resolution HLA typing methods were applied or data for specific loci are missing. One can infer the likelihood of specific high-resolution genotypes, here meaning an HLA genotype with two-field allele names based on standard HLA nomenclature (Marsh, 2022), from incomplete or low-resolution data by comparing known HLA sequences (Barker et al., 2023) and by utilizing patterns in genotype and haplotype frequencies (Maiers et al., 2007; Gragert et al., 2013). While each HLA allele (e.g*.*, DRB1*15:01) corresponds to multiple distinct nucleotide sequences, for epitope analysis we focus on distinct protein sequences and disregard synonymous nucleotide sequences and variation within intronic and UTR sequences. Following this definition, an HLA haplotype consists of a set of select two-field HLA alleles on a single copy of chromosome 6 (e.g., A*11:01∼C*12:03∼B*27:12∼DRB1*16:01∼DQB1*05:02) (Mack et al., 2023). By using population haplotype frequency tables, a set of high-resolution genotypes is estimated from low-resolution (e.g*.*, DRB1*15) data. The available haplotype frequencies can vary widely based on the individual’s self-reported race/ethnicity/ancestry information. Consequently, providing this information improves the accuracy of imputation (Dilthey et al., 2013; Hollenbach et al., 2015; Israeli et al., 2023; Madbouly and Bolon, 2024).

Imputation algorithms are probability-focused techniques. They carry inherent risks, such as the possibility of incorrectly assigning a genotype based on probability assumptions. These risks may be greater if ancestry information is not available, cannot be practically or ethically applied, or if a self-identified ancestry does not completely reflect their genetic ancestry. These risks are compounded in admixed populations, where an individual’s two HLA haplotypes may not be derived from the same origin population (Maiers et al., 2013). Moreover, in the context of transplantation, it may be necessary to impute the recipient and donor typing, or both, which multiplies the number of combinations, and adds more uncertainty when comparing self and non-self HLA.

Imputation can follow distinct strategies (Li et al., 2015), each with corresponding advantages and disadvantages. Perhaps the simplest of strategies in the HLA context is single imputation. In this strategy, an HLA genotype prediction from a single run of imputation is selected. Most strategies opt for selecting the most likely HLA genotype based on the most likely combination of haplotypes matching the observed low-resolution data. This approach is commonly used in the context of HLA imputation (Ferradji et al., 2017; D’Souza et al., 2018), although selecting the most likely option may create a false precision, where a single genotype is selected more frequently than it is expected to occur in a population. Another variant of single imputation is to assign a single predicted genotype by using a weighted random selection from candidate haplotype pairs, which reflect their observed frequencies within a population. This approach has the advantage of reducing the bias of single imputation at the population level but may introduce random errors for individuals.

Multiple imputation in the HLA context refers to a process of repeatedly imputing from weighted distributions to calculate a set of multiple options with corresponding likelihoods, in contrast to selecting a single “winner-takes-all” option. There are many strategies to perform multiple imputation, and in the context of HLA, the multiple imputation options can be calculated by sampling from weighted population frequencies or by brute force analysis of a complete list, with an optional minimum frequency threshold. Multiple imputation can become more accurate with deeper sampling of options (Louzoun et al., 2018), but with the wide variety observed in HLA haplotypes (Gragert et al., 2013) considering all possible haplotype combinations is a challenge. One popular tool used for multiple imputation is the NMDP HaploStats application (Madbouly et al., 2014) (www.haplostats.org). This platform considers all possible known haplotype combinations, provides insights on expected genotype frequencies and Hardy Weinberg equilibrium, and has potential use in epitope studies (Krummey and Cliff Sullivan, 2022).

In situations where a single genotype is not necessary, such as the case of analysis of quantitative epitope prediction, an approach that aggregates a number of likely haplotype combinations simultaneously can be considered. Algorithms considering these multiple combinations need to aggregate epitope analysis of transplant pairs by, e.g*.*, using population frequencies as weights. When these weighted genotype combinations are analyzed in molecular matching algorithms, these weights (or summed weights) can also be applied to the corresponding predicted epitope scores, thereby providing a summed epitope score (Geneugelijk et al., 2017).

The present study aimed to quantify the differences in epitope predictions from genotypes derived from ancestry-based imputation. We used simulated and real-world patient and donor groups with high-resolution HLA genotyping. Single and multiple imputation algorithms implemented in the PIRCHE prediction pipeline (Geneugelijk et al., 2017) were applied considering haplotype frequencies corresponding to different ancestries. The imputed genotypes were analyzed for the number of predicted indirectly recognizable T cell epitopes (PIRCHE score) (Geneugelijk and Spierings, 2020) and surface-exposed amino acid mismatches (Snow score) (Niemann et al., 2022; Niemann et al., 2023). These imputations and epitope predictions were repeated with multiple assumptions on their ancestry. Aggregated epitope scores were compared between ancestry assumptions, and between the contexts of single and multiple imputation.

As shown previously (Geneugelijk et al., 2017), we hypothesized that a majority of transplant cases will only have small deviations between true molecular matching scores calculated based on high-resolution genotyping data and scores calculated through imputation. In cases with incorrectly configured self-reported race/ethnicity/ancestry, we hypothesized increased discrepancy that may have limited clinical impact in most transplant cases considering previously defined thresholds.

## Methods

### Simulated patient and donor groups

Simulated high-resolution genotypes were generated based on 2011 NMDP haplotype frequencies (Gragert et al., 2013). The frequencies in this dataset are divided into five broad categories and subdivided into 21 sub-categories. Each of these categories represents a distinct population (Supplementary Figure S1). Furthermore, a “SUPER-population” was artificially generated for use in imputation by aggregating all available haplotypes and accumulating the haplotype frequencies and assigning a normalized weight based on their relative frequencies within each group.

For each of the 21 available ancestry sub-categories, a sample dataset of 1,000 simulated individuals were generated for both the patient and donor datasets. These individuals were simulated by a weighted random selection of two haplotypes, where the likelihood of selecting a haplotype corresponds to that haplotype’s reported frequency. By pairing two haplotypes together, we create a simulated individual’s HLA genotype, and an expected frequency within their corresponding population calculated as the product of the respective haplotypes’ reported frequencies. The assumed ancestry of each simulated patient or donor is assigned to match the population frequency dataset from which their haplotypes are derived. These population-specific patient and donor datasets were paired in a stratified manner across and within ancestry groups. This stratification provided a “randomly paired” dataset that mimics randomly allocated solid organ transplantations within populations, as well as across populations. As the source ancestry in generated genotypes is known, we used this as a reference ancestry in downstream analysis.

Low-resolution (group-level) typings were derived from the simulated high-resolution genotypes. To this end, two-field HLA allele names (Marsh, 2022) were reduced to the first field of nomenclature to yield allele-group level genotypes. In high-resolution “control” and low-resolution cases, PIRCHE high-throughput input files were generated in the standard paired patient-donor. csv format for subsequent analysis. Serological typing resolution (Kaur et al., 2018; Osoegawa et al., 2022) was not considered in this analysis.

### Real patient and donor groups

As a confirmatory dataset to supplement simulated patient and donor groups, supplementary analysis was performed on two datasets of real-world transplantation data. All transplant samples were anonymized and had available genotype data for *HLA-A, -B, -C, -DRB1* and *-DQB1* at the two-field allele-level. 439 transplantations carried out at the Universitätsspital Basel were selected (ethics committee approval number 2023–01992). In the Basel dataset, ancestry data was not provided, and all patients and donors were therefore assumed to be European Caucasian (EURCAU) ancestry. A dataset from the University of North Carolina at Chapel Hill (UNC) consisting of 9,471 samples with high-resolution HLA genotyping was also analyzed. The UNC data contained self-identified race information using broad ancestry categorizations (Supplementary Figure S2), but did not specify patient and donor pairing. Thus, the samples were randomly paired into 4,735 patient-donor pairs.

Similar to the approach to simulated genotypes, low-resolution genotype datasets are generated by replacing each allele from the high-resolution genotypes with its corresponding 1-field allele group, separately for transplant patients and donors. The low-resolution and high-resolution genotypes were analyzed using the PIRCHE and SNOW modules, with imputation carried out using the 27 specific and broad ancestry assumptions.

### Imputation

High-resolution (two-field allele-level) genotypes were imputed from the simulated low-resolution genotypes. Since genotypes are generated using locus-specific genotyping methods, haplotype definitions are not directly known. One multiple imputation strategy has been suggested (Geneugelijk et al., 2017) by forming all possible combinations of alleles to form pairs of haplotypes corresponding to the provided genotype (Supplementary Figure S3). These haplotype pairs were then searched for in the NMDP haplotype frequency tables (Gragert et al., 2013), by considering the alleles’ respective first field. In case no matching haplotypes are identified in the NMDP tables, linkage assumptions between HLA loci are removed stepwise (Supplementary Figure S4). This fallback approach is penalized with lower frequency weights to favor combinations of natural haplotypes. A genotype’s frequency is defined as the product of its corresponding haplotype frequencies. Frequencies of all identified genotypes are normalized and to limit computational effort, only the top 99% most frequent genotypes are considered in multiple imputation.

For each of the identified genotypes, molecular matching is performed. Scores from multiple imputation options are aggregated based on a weighted average, where weights correspond to the imputed genotype frequencies (i.e., aggregated multiple imputation). The aggregated multiple imputation algorithm is incorporated in the PIRCHE webservice (www.pirche.com). The imputation algorithm is automatically applied on the input low-resolution HLA genotypes when using the PIRCHE bulk. csv analysis tool. The protocol describing bulk. csv PIRCHE analysis and data processing was previously described (Niemann et al., 2024).

To simulate single imputation, the same method was applied, but only the single most frequent genotype was identified and used in downstream analysis. Single imputation within the PIRCHE service is facilitated by extracting imputed genotypes first and only considering the most likely genotype results in the analysis.

### Strict imputation

Parallel to that, the above imputation was additionally applied without the previously described fallback approach in case of absent matching haplotypes (*i.e.*, strict aggregated multiple imputation). Instead, in these cases when no haplotype combinations are found, imputation was aborted and no result was calculated.

### Allele-level mismatches

The high-resolution ground-truth genotypes were compared to those generated by single-imputation. In each case where a two-field imputed genotype does not match the original high-resolution genotype, the allele mismatch was quantified and summarized. Allele-level mismatches were calculated separately for imputation in each ancestry assumption.

### Molecular matching

As representative molecular matching algorithms, the PIRCHE and the Snow algorithms were applied. PIRCHE represents the prediction of indirect T cell epitopes. The PIRCHE-II score is calculated as previously described (Geneugelijk and Spierings, 2020). In brief, donor HLA-derived peptides likely to be presented by the recipients DRB1 that are not present in the presented self-peptidome are considered as PIRCHE. The number of such distinct core peptide:HLA tuples is considered the PIRCHE score.

The recently described Snow algorithm is considered as an approach for antibody epitope matching. In brief, Snow identifies the number of surface-exposed amino acid mismatches between patient and donor. For that purpose, Snow evaluates HLA protein-specific solvent-accessible surface area (Niemann et al., 2022) and local ellipsoid protrusion (Niemann et al., 2023).

In the case of imputed genotypes, PIRCHE-II and Snow molecular mismatch loads are calculated by summing predicted mismatch loads across all multiple-imputed genotype pairs. An aggregated score is calculated by multiplying epitope scores with weights corresponding to normalized predicted genotype frequencies.

### Performance evaluation

**FIGURE 1:**
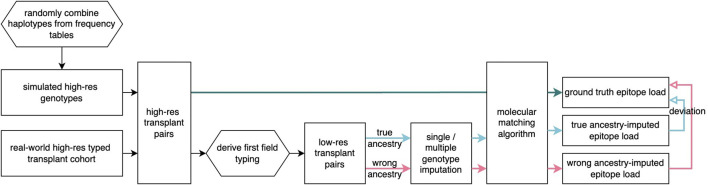
Flowchart of imputation analysis Simulated patient and donor groups are generated and real-world transplant pairs are selected and prepared at two-field resolution at five loci. From these, the first-field genotypes are extracted, and imputation methods are applied with varying ancestry assumptions. In all cases, molecular matching scores were found and compared across ancestry assumptions.

For the virtual and real transplant datasets, high-resolution genotypes were considered for calculation of ground-truth molecular mismatch loads. The molecular mismatch score calculated for each patient-donor pair on a high-resolution genotype provides an unambiguous baseline score, which is subsequently used as a reference to calculate deviance introduced by imputation in each population context. Using the respective low-resolution genotypes, molecular matching scores were calculated considering different imputation approaches using the 21 detailed ancestry groups, five broad ancestry categories and the SUPER-population. The differences between imputed and ground-truth scores were considered as deviation (Figure 1). Log-transformed delta scores are calculated to compare the ground-truth molecular mismatch load with the estimated scores derived from imputed genotypes to quantify differences in predicted scores.

Each patient or donor in the simulated and real-world datasets has an assumed ground-truth ancestry (either self-identified or assigned in the case of Basel dataset). Analyses were grouped by ground-truth ancestry and imputation ancestry to evaluate ancestry-specific deviations introduced by imputation. The ground-truth ancestry is compared against the specific or broad frequency category used in imputation to assign an imputation categorization “match” or “mismatch”. Additionally, the imputation performance based on allele mismatch was evaluated with different statistical metrics including the area-under-curve (AUC) of receiver-operating-characteristic curve score (Hanley and McNeil, 1982), Brier score (*i.e.*, mean-squared-error) (Brier, 1950; Steyerberg et al., 2010), weighted F1-score (*i.e.*, harmonic mean of precision and recall) (Chinchor, 1992) and balanced accuracy (*i.e.*, the arithmetic mean of sensitivity and specificity) (Supplementary Figure S5).

## Results

### Imputation success depends on ancestry assumption

**FIGURE 2:**
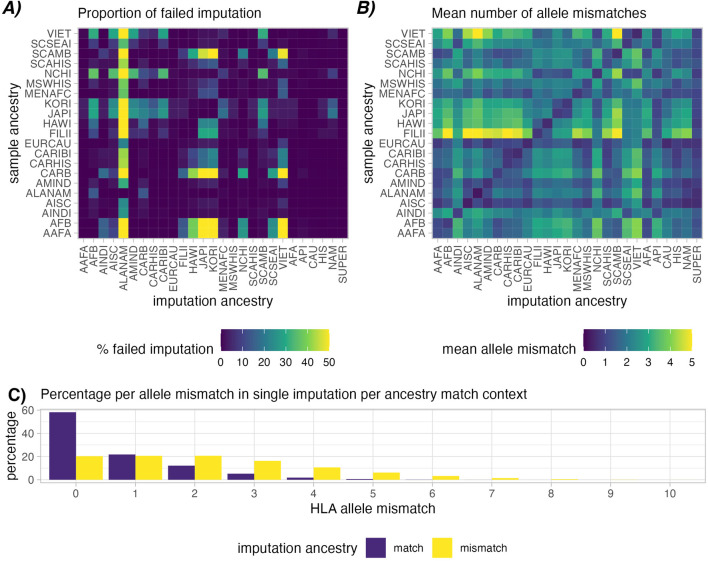
Imputation success in simulated patient and donor groups. Heatmap **(A)** shows relative rates of failed imputation, where valid haplotype pairs were not found based on ancestry assumptions (lower is better). This is most common when using the ALANAM haplotype dataset for imputation. Heatmap **(B)** shows mean allele-level mismatches between the high-resolution genotype and the successfully single imputed genotype (lower is better). A higher degree of matching can be observed in cases with matched sample and imputation ancestry (Diagonal). Correspondingly, panel **(C)** provides a histogram of allele mismatch, distinguishing matched, i.e., correct ancestry assumption (purple) and mismatched, i.e., incorrect ancestry assumption (yellow), the latter showing a clear shift towards higher proportions of HLA mismatches.

Imputation of high-resolution 5-locus genotypes from low-resolution genotypes presents inherent risk of inaccuracies (Figure 2). This error is categorized by A) proportion of cases where valid combination of HLA haplotypes could not be found in the haplotype frequencies (“failed” imputation, Figure 2A), and B) allele mismatches between the ground-truth high-resolution genotype and the imputed genotype (Figure 2B).

Imputation success was observed to vary depending on ancestry assumptions during imputation. In the simulated patient and donor groups, imputation was successful in ∼99.98% of the cases when the imputation population matched the sample ancestry (Figure 2A Diagonal). Imputing genotypes with incorrect ancestry assumption however we observed an average ∼92.95% success rate. ALANAM haplotypes were observed to most frequently fail to impute valid genotypes, which is also the ancestry category with the fewest listed haplotype frequencies (138) in the final 2011 dataset (Gragert et al., 2013), corresponding to the lowest sample size used in frequency estimation (1,376). Strict imputation is expectedly more susceptible to imputation failure, with an observed average of 91.99% successful imputations in case of correctly assumed ancestry and 45.95% successful imputation in case of incorrectly assumed ancestry (Supplementary Figure S6A).

Similar patterns were observed in the mean number of allele mismatches in single imputation. When imputation ancestry was matched with sample ancestry, we observed a lower mean allele mismatch count compared with imputations with mismatched ancestry (0.73 vs. 2.19, Figures 2B, C). We discovered that mean allele mismatches are highest (mean = 5.36) in the case of Filipino (FILII) individuals imputed based on American Indian South or Central America (AISC) ancestry assumptions. In strict single imputation, although imputation failed more frequently, we observe comparatively fewer mean allele mismatches (0.62 in the ancestry matched and 1.39 in ancestry mismatched, Supplementary Figure S6B).

When considering the extra evaluation metrics (Supplementary Figure S5), The Filipino (FILII) category suffers the most in general from wrongly assumed ancestry when looking at the F1-score (mean = 0.72), Brier score (mean = 0.41), balanced accuracy (mean = 0.52) and AUC score (mean = 0.81). This corresponds to the observations shown in Figure 4 of Gragert et al. (2013) where haplotype frequencies for FILII show the least similarity with other US populations as well as other API populations. In general, the categories that fall within the API broad category, which contains the FILII specific group, are quite genetically distinct from other populations and appear to suffer the worst from wrongly assumed ancestry.

### Accuracy of epitope analyses depends on ancestry assumption

We evaluated deviations of multiple imputed PIRCHE and Snow scores to evaluate the imputation accuracy in the context of molecular mismatch analyses. Analysis was focused on simulated datasets where only one party per transplant pair was imputed, with the respective other individual keeping its true high-resolution genotype. This allows more specific visualization and allows us to assess the role of inaccuracies in either recipient or donor imputation.

**FIGURE 3:**
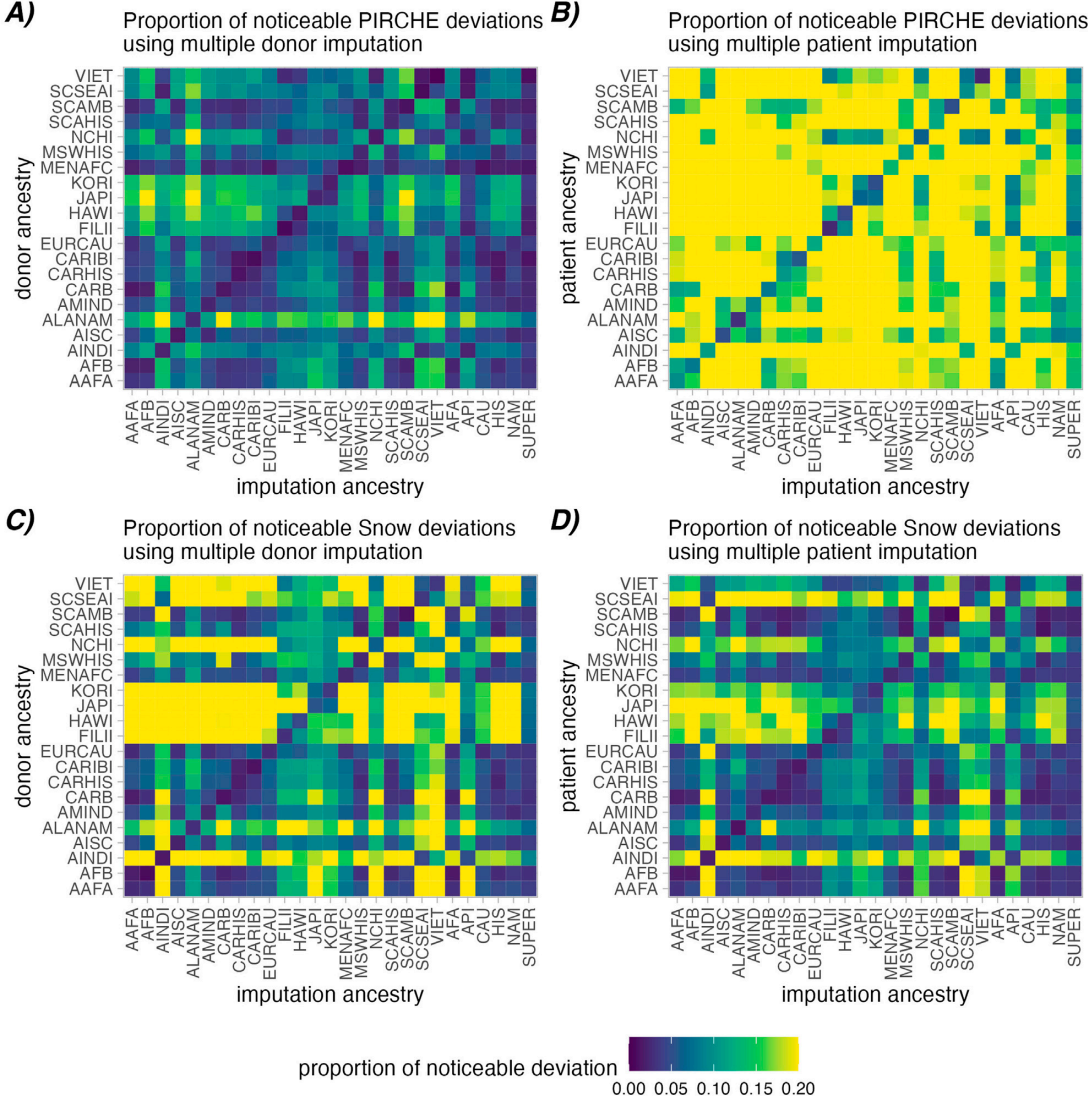
Proportions of noticeable molecular mismatch deviations between high-resolution and aggregated multiple imputed molecular matching scores. A noticeable deviation is defined as a deviation in the Ln (PIRCHE-II) **(A, B)** or Ln (Snow) scores **(C, D)** greater than 0.1. Deviations introduced by imputation of donor genotype is shown in **(A, C)**, while imputation of the patient genotype is shown in **(B, D)**. Heatmap cells show the proportion of cases that have noticeable deviations across ancestry assumptions (lower is better).

In this analysis, molecular mismatch loads (PIRCHE-II and Snow) were calculated in high-resolution pairs and in the imputed pairs. The log deviations (e.g., Ln (PIRCHE-II High Res) - Ln (PIRCHE-II Multiple Imputed)) were calculated in every case, and noticeable deviations were defined as cases which exceeded a log-deviation of 0.1. Figure 3 indicates the proportions of such cases for the simulated patient and donor groups where donors (Figures 3A, C) or patients (Figures 3B, D) were multiple imputed. The simulated patient and donor groups consider individuals derived from 21 sub-populations in the 2011 NMDP haplotypes frequencies. Imputation was performed using the same 21 sub-populations, as well as five broad categories and the derived SUPER-population.

As observed in Figure 3 (Diagonal), aggregated multiple imputation scores had a lower proportion of noticeably deviating PIRCHE-II and Snow scores in all cases where imputation ancestry matched the true ancestry vs. mismatched (PIRCHE-II: 1.7% vs. 8.6% for donors, 7.8% vs. 23.5% for patients; Snow: 2.8% vs. 14.5% for donors, 2.4% vs. 10.8% for patients). Similar patterns were observed in the single imputed context and in strict analysis (Supplementary Figures S6–S10). Interestingly, single imputation resulted in a slightly higher proportion of noticeably deviating results (PIRCHE-II: 2.9% vs. 1.7 for donors, 8.0% vs. 7.8% for patients; Snow: 4.1% vs. 2.8% for donors, 3.3% vs. 2.4% for patients). When comparing the aggregated strict multiple imputations, where only naturally occurring haplotype frequencies were considered, had a slightly lower proportion of noticeable molecular mismatch load deviations in the successful imputations (PIRCHE-II: 1.5% vs. 1.7% for donors, 7.2% vs. 7.8% for patients; Snow: 2.1% vs. 2.8% for donors, 2.6% vs. 2.4% for patients).

Generally, imputation of patients appears to be more susceptible to deviations in PIRCHE-II score than imputation of donors. Conversely, imputation of the donor has a greater effect on calculating the Snow scores compared to imputing the patient genotype. Furthermore, imputation using the evenly distributed SUPER-population had the lowest average proportion of noticeable deviations in molecular matching scores accumulated across all individual ancestry, followed by the CAU broad ancestry category, indicating an added value of large sample size in the quality of estimated haplotype frequencies.

### Risk stratification deviates depending on ancestry assumption

**FIGURE 4:**
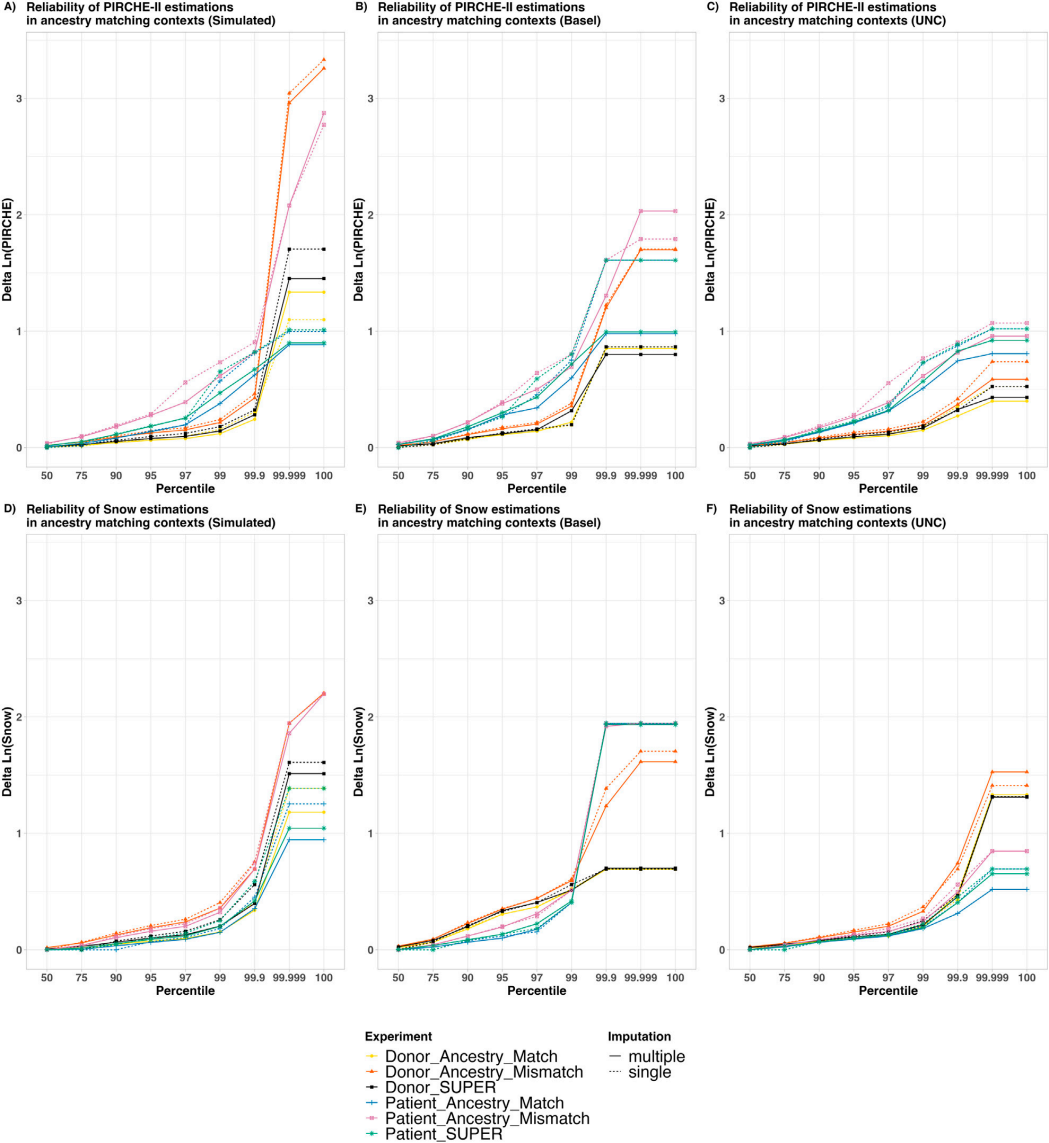
The Reliability of PIRCHE-II and Snow estimations in varying ancestry matching contexts. The X axis shows an overview of the patient and donor groups stratified by cumulative percentile, with a Y axis showing the Delta Natural Log (PIRCHE-II) or Delta Natural Log (Snow). Colors represent whether patients and/or donors are being imputed and whether or not the imputation ancestry matches the individuals’ ancestry. The solid and dashed lines show contrast between aggregated multiple imputation against single imputation. This represents a comparison of maximum epitope error risk across a sample in varying imputation contexts. The top panels **(A–C)** show Ln (PIRCHE-II) deltas (T cell epitopes), and the bottom panels **(D–F)** show Ln (Snow) deltas (B cell epitopes). Reliability plots are shown for simulated datasets with separated Patient and Donor imputations **(A, D)**, real world datasets from Basel **(B, E)** and real-world UNC Data **(C, F)**. No self-identified ancestry data was available for the Basel dataset, and all samples were assumed to fit the “European Caucasian” categorization. In the UNC dataset, self-identified ancestry was provided in the five broad categories, and the relative numbers of samples are shown in Supplementary Figure S2.

To further categorize how much risk can be expected based on the incorrect ancestry assumptions, these deviations were plotted in the risk plots of Figures 4A–F. In these plots, the Y axis represents a calculated deviation in the Ln (PIRCHE-II) or Ln (Snow) scores, while the X axis is cumulative percentiles across the sample of patient and donor pairs. Lines were plotted to show which proportion of the samples falls below a certain risk threshold of risk for varying imputation contexts.

Several observations were made based on this data. First, we observed that the “Ancestry Match” lines (Yellow and Blue) lie lower than their corresponding “Ancestry Mismatch” lines (Orange and Pink, respectively), suggesting that less error was introduced when the correct ancestry is assumed. Furthermore, we observed that the error introduced by using the SUPER-population in imputation for donors (Black) and for patients (Green) lies between their corresponding “Ancestry Match” and “Ancestry Mismatch” lines. This indicates the imputation using the SUPER-population introduced more error than using the “matched” ancestry, but less error than using the “mismatched” ancestry.

In all cases, for the 50% percentile, the maximum observed deviation was less than 0.1, indicating that for at least half of the sample there was no noticeable deviation in epitope score. Furthermore, in nearly all contexts, for the 99% percentile categories, delta scores were well below a delta of 1.0, which notably has been used as a threshold where epitope scores may be considered as a separate risk category (Geneugelijk et al., 2017). We did however observe in the higher percentiles some cases with greater variability between ground-truth epitope scores and scores from imputed genotypes, which are explained below.

Regarding imputation algorithms, we observed that deviation scores in general are higher in single imputation (dashed lines) compared with their corresponding aggregated multiple imputation (solid lines) of the same color. This indicates that in general the multiple imputed scores more closely matched the high-resolution scores than single imputed scores.

When contrasting T cell (Panels A–C) and B cell epitope analysis (Panels D–F), we made similar observations to those from Figure 3. In the PIRCHE-II context, imputation of the patient genotype (Blue, Pink, Green) was observed to lead to deviations from the ground-truth in their corresponding contexts than donor imputation (Yellow, Orange, Black, respectively). And again, conversely, imputation of the donor genotype was more likely to lead to deviations in Snow scores; although in the simulated dataset, ancestry match seems to play a more important role. In the cases where the patient and the donor are both imputed (Supplementary Figure S11), we observed generally higher deviations across the population compared to cases where only one of the two are imputed. This supports the hypothesis that imputing both patient and donor genotypes may lead to more error than imputing a single genotype.

There are a few outliers which can be noted in the 99.999 and 100 percentile, leading to the crossing of imputation context lines. It should be noted that these are very rare cases, and the cause of these deviations can be attributed to one of two situations: 1) The ground-truth patient is *HLA-DRB1* homozygous but their corresponding low-resolution genotype was erroneously re-imputed as *HLA-DRB1* heterozygous (or vice-versa). Alternatively, 2) when the ground-truth epitope score is 0, but the imputed score is imputed as a low but non-zero. These miniscule differences in molecular mismatch score, when log transformed, can appear as drastic log-differentials.

## Discussion

### Imputation and self-identified ancestry

HLA genotype imputation is a controversial topic in molecular HLA matching. Geneugelijk *et al.* reported a limited clinical impact of the uncertainty introduced by multiple imputation, considering a patient cohort mostly of European descent (Geneugelijk et al., 2017). Opposed to that however, D’Souza *et al.* report considerable deviations in molecular matching scores using single imputation using most likely predictions through Haplostats (D’Souza et al., 2018). A similar approach was used in analysis of Class II HLA-derived AB-verified eplet scores by Renuncio-García et al. (2022). In a cohort of 419 kidney transplant patients, Crane et al. (2024) suggested high-resolution typing as a prerequisite for molecular matching given the PIRCHE-II score deviations found in their cohort. These studies suggest that imputation is a promising approach to analyze ambiguous molecular matching data, so long as care is taken and certain restrictions are held. Imputation approaches were successfully applied in large-scale retrospective cohorts to enable molecular matching analyses, yet for specific cases, imputation may yield high deviations from the true molecular match grade. As typing methods advance, real-time protein-level analysis can be performed to confirm and refine the observations made here.

It is important to consider that self-identified race/ethnicity/ancestry is not fool-proof and can introduce human error and subsequent challenges in understanding patterns in HLA genotypes and haplotypes (Hollenbach et al., 2015). Ancestry may be intentionally or unintentionally incompletely represented when it is self-reported. This is an important consideration when choosing a reference ancestry for comparison and identifying deviations from a reference epitope score. Thus, a reference ancestry as it is used in this study is not to be considered as a “correct” ancestry, but as a baseline for comparison. This is similar to the concept of identifying genomic variance from a reference sequence, where the reference is not necessarily more “correct” than the analyzed variant.

We should note that the ancestry categorizations used within this study and within the NMDP haplotype frequencies (Gragert et al., 2013) are imperfect and do not fully capture the patterns in genetic ancestry. Furthermore, these categorizations are not to be considered an absolute truth or reflection of discrete differences in populations. These categorizations may not reflect well-defined population groups which adhere to commonly-accepted qualities for use in statistical analysis such as random mating and Hardy-Weinberg equilibrium (Chen et al., 1999). The simulated and real-world patient and donor datasets do not represent any larger populations, and population-level observations cannot be made based on these datasets. Many of the ancestry categorizations used within this study, such as “caucasian”, have been made obsolete and are no longer appropriate as the common use deviates from their original definition of originating from the Caucasus (Gombault et al., 2023). Other studies, such as the 1,000 genomes project (Abi-Rached et al., 2018) use a distinct system of genetic ancestry categorization, which if applied in an imputation context could result in slightly varying conclusions and interpretations. Comparing and contrasting these ancestry categorizations and assessing these categories in, e.g*.*, epitope analysis based on imputation across these categories would be an interesting followup study.

How to most effectively and ethically use ancestry categorizations in transplantation is an ongoing discussion. This is especially true within the NMDP, which periodically updates their definitions as seen in the revision in the major definitions of SIRE categorizations on donor recruitment forms in 2020 (Madbouly and Bolon, 2024). It is critical to use care and exercise caution when using these categorizations, and to keep in mind the ultimate aim of providing equivalent access to medical care for all individuals.

As an alternative to imputation using a self-identified ancestry categorization, it would be interesting to perform imputation based on an assessed genomic ancestry category. This predicted categorization could come from an HLA imputation algorithm itself, as demonstrated in Israeli et al. (2023), or alternatively from a separate test to determine genetic ancestry based on a panel of polymorphism. Using, e.g*.*, a panel of SNPs to directly determine an individual’s genetic ancestry could alleviate some human error involved with self-identification, and possibly categorize individuals from admixed ancestry, as well as provide assessment in cases where self-identified ancestry cannot be practically, legally, or ethically obtained. However, this assessment would introduce a separate set of ethical, consent, practical, or financial challenges. To our knowledge, a curated dataset directly correlating population-specific HLA haplotype frequencies with directly-assessed genetic ancestry does not yet exist, and can therefore not be practically applied in imputation algorithms. In any case, directly assessing an individual’s genetic ancestry would need to be undertaken only after consideration of the ethical considerations.

### Imputation quality

Depending on the ancestry used in imputation and whether or not it matches the ground-truth ancestry, we observed a high variance in rates of successful imputation (Figure 2A). However, we also observed a poor success rate for ALANAM, a population dataset for which very few haplotype frequencies are provided. Notably, frequencies are provided for only 138 distinct haplotypes (compared with, e.g*.*, 37,645 available haplotype frequencies for CAU), which reflects the very low sample size from which these frequencies were derived (1,376 compared with, e.g*.*, 3,912,440 individuals used in estimating CAU frequencies) (Gragert et al., 2013). The difficulty in imputing based on this limited dataset suggests that for individuals that are under-represented in the haplotype frequencies, imputations based on broad groups may provide more helpful genotype predictions. Specifically, in the case of ALANAM or other population groups with lower representation in the haplotype frequencies, a more accurate risk estimation could be made by assuming a broad NAM population, which was generated based on 46,147 individuals.

Similar conclusions can be drawn when comparing mean allele mismatches from relatively distinct ancestry groups. Notably, we observed the highest mean allele mismatch counts when Filipino individuals were imputed based on American Indian South or Central America assumptions. This likely reflects a large genetic distance between these two populations, as was previously reported in Gragert et al. (2013) in the Nei’s Genetic Distance plot (Figure 2) and Kendall Rank Correlation plot (Figure 4). This could indicate that imputation based on more closely related (but still mismatched) populations might be more successful or accurate as opposed to distant and distinct populations. Categorizing imputation quality and scaling by population genetic distance could be an interesting followup study.

### Epitope deviations

An interesting conclusion from Figures 3, 4 was that in the context of PIRCHE-II, imputation of the patient genotype resulted in more deviation than imputation of the donor genotype. This conclusion is logical, because PIRCHE-II T cell epitopes are defined by (donor-derived) peptides predicted to be presented in the context of a (patient-derived) presenter HLA-DRB1. If a (patient) *HLA-DRB1* allele is “incorrectly” imputed and assessed, the erroneous *HLA-DRB1* may predict a distinct potential peptidome and have a notable effect on the resulting PIRCHE-II score. However, when a single allele from the donor is “incorrectly” imputed and assessed, we would expect a comparatively smaller number of potentially presented peptides. This suggests that the availability of a patient high-resolution genotype is more important in PIRCHE-II analysis than the availability of a high-resolution donor genotype.

Conversely, we observed in Figures 3, 4 that imputed donor genotypes had more of an effect on deviation in the context of Snow B cell epitopes. This is also intuitive, as we define B cell epitopes by predicted antibody-accessible amino acids on the surface of the (donor-derived) HLA. Self-epitopes are only subtracted from a molecular mismatch score if they are also found on the donor HLA, so an “incorrectly” imputed and assessed patient-derived HLA would be less likely to affect the predicted molecular mismatch load. The suggestion here is that in Snow B cell analysis, the availability of donor high-resolution genotype is more important than availability of high-resolution patient genotype.

In Figure 4 we saw that there were rare cases in the single imputation figures. In these cases of apparently high deviation, we looked for the cause, and found that it can be explained by one of two situations. In the first scenario, a very small differential is amplified during the logarithmic transformation. In one specific case, the high-resolution typing as simulated was a 9/10 match, wherein every *HLA-A*, *-B, -C*, and *-DRB1* allele matches. The only difference was in *HLA-DQB1*, where a patient and donor had a DQB1*06:02 vs. DQB1*06:04 mismatch. By reducing the genotypes to allele group level and re-imputing, the resulting single-imputed genotypes are identical. Therefore, imputation results in a slightly different PIRCHE-II score; In the high-resolution case we saw a PIRCHE score of 5, while in single imputation we see a score of 0. Both of these scores are quite low and are not likely to result in a different risk categorization. But the difference is somewhat drastically amplified in the logarithmic Ln (PIRCHE) differential. This suggests that in rare cases the process of single imputation can lead to single allele discrepancies and care should be taken in interpretation.

A second scenario was identified where the patient’s homozygosity or heterozygosity can result in deviation molecular mismatch scores. PIRCHE scores are derived from a count of potential T cell epitopes. We consider a single distinct T cell epitope as a presenter HLA combined with a putative presented peptide. In the case of homozygous *HLA-DRB1* individuals, there are a lower number of distinct presenter HLAs compared with a heterozygous individual, and epitopes defined by the two identical homozygous presenters are not counted separately. This could indeed affect comparability across clinical cases. This is a common challenge among molecular matching algorithms, especially in imputed PIRCHE, where uncertainty may be identified at multiple stages. In the edge cases in Figure 4A with the highest deviation, the difference can be attributed to patients who are ground-truth *HLA-DRB1* homozygous, but erroneously imputed to be *HLA-DRB1* heterozygous (or vice-versa), resulting in a log differential reflecting approximately double (or half) the PIRCHE score. The impact of heterozygosity and homozygosity in molecular matching context is to be explored further in future studies.

A somewhat surprising observation was in the combined SUPER dataset in Figures 2, 3. This set is created by combining, with equal weight, haplotype frequencies from all 21 of the split ancestry categories. Although this dataset does not nearly represent any real-world population, it does have a wide variety of available haplotypes which can be used in imputation. With such a wide variety, it was successful in 100% of imputations (Supplementary Figure S6A), but resulted in an average of 1.03 allele mismatches (compared with the 0.73 observed in ancestry-matched imputation). The CAU broad population also performed quite well in imputation success with similar drawbacks in allele-level mismatches. This suggests that in the absence of a known ancestry, or in situations or countries where ancestry information cannot be practically or ethically collected, SUPER could be a viable assumption, and otherwise CAU. However, since this is a flawed assumption, and especially based on the equally-distributed ancestry frequencies, this would introduce risk and more reliable conclusions could be made by using a correctly assessed ancestry.

### Limitations

This study has several limitations. Only 5 HLA loci are considered and assessed in this analysis, and T cell epitopes are predicted only in the context of a presenter HLA-DRB1. Other HLA loci are certainly important in transplantation (Daniëls et al., 2021), and polymorphism across the MHC including all HLA loci could help to define haplotype patterns (Matern et al., 2020). Furthermore, imputing ancestry of human individuals will need to be considered regarding ethics and investigated regarding IRB regulations.

The haplotype frequencies used in this study are based on the 2011 NMDP dataset (Gragert et al., 2013). This dataset was selected due to its non-volatility and because of its common use within the field. However, there may be value in using a more recent or complete dataset. One alternative would be to use a broader set of allele frequencies from, e.g*.*, allelefrequencies.net (Gonzalez-Galarza et al., 2020) which collects and provides global HLA allele and haplotype frequencies. But there remains a challenge in collecting and curating this data, which exists at varying HLA genotype resolutions, and unstandardized definitions of ancestry or geography. But it remains a challenge to collect and curate the data at varying resolutions. Another idea would be to use frequency data from alternative polymorphism datasets such as the 1,000 genomes project (Fairley et al., 2020), which categorizes polymorphism in terms of population frequency. While HLA information can be derived or assessed for the samples in this project (Gourraud et al., 2014; Abi-Rached et al., 2018), it likely is not suitable for use in analysis of ancestry-specific HLA haplotype frequencies used in imputation, as the lower sample sizes and targeted sample inclusion would not be able to generate haplotype frequencies that reflect real-world donor and patient populations.

Several other imputation contexts could be considered and compared against imputation from first-field genotypes. For example, this study could be extended by imputing a missing genotype at a given locus. Another strategy would be to reduce our ground-truth genotypes to antigenic equivalents or NMDP multiple allele (MAC) codes, rather than first-field group-level genotypes. And a third strategy would be, in the single imputation approach, to randomly sample a genotype prediction from the full imputation output instead of selecting the most likely genotype prediction. In all of these contexts, we might expect a different range of differential scores, but would likely come to similar conclusions regarding ancestry assumptions.

## Conclusion

To the best of our knowledge, our study is the first to compare uncertainty introduced by single and multiple imputation in the context of molecular HLA matching and to quantify error introduced by unknown or incorrectly configured ancestry. It confirms earlier observations specific to a European cohort and is extended to more global heterogeneous cohorts, as well as real-world populations, and builds on previous observations on limited impact of imputation. We have shown that choosing a population frequency dataset for imputation that matches the ancestry of a patient or donor, compared to an unmatched ancestry, can lead to more accurate imputation results and epitope analysis. We have also shown that the use of multiple imputation can lead to a more balanced analysis with lower rates of error in epitope analysis compared with single imputation.

The comparatively low failure rates in strict imputation is encouraging, and indicates that the use of adaptive imputation strategies (Supplementary Figure S4) is a promising approach. It further suggests that imputation by analyzing blocks of the MHC in stronger linkage disequilibrium is a reasonable approach to predict most likely genotype combinations and suggests a need for refined imputation algorithms. While reducing to known haplotypes from subsets of the genotypes provides one shortcut to haplotype estimation, there is room for improvement. The combined SUPER-population does show promising results for a first-pass imputation strategy but should be refined to find more likely haplotype combinations to more accurately predict resulting genotypes.

These results reinforce that the use of high-resolution genotypes will result in more accurate epitope predictions, and should be used as a best-case solution, especially in cases where ancestry data cannot be legally collected. But in the absence of high-resolution genotypes in a vast majority of cases, the epitope scores will be similar and does not result in clinically relevant distinct risk categorization. Furthermore, this analysis can only be performed with the availability of complete and high-quality data, and further reinforces the value of providing and collecting self-reported race, ethnicity, and ancestry data (Madbouly and Bolon, 2024).

## Data Availability

The original contributions presented in the study are included in the article/Supplementary Material, further inquiries can be directed to the corresponding author.

## Supplementary material

SUPPLEMENTARY FIGURE S1 Twenty-one detailed ancestry categories and descriptions and five broad ancestry categories were considered as suggested by Gragert et al. (2013). In parentheses is the number of individuals from which these haplotype frequencies were generated, as shown in Tables 1, 2 of Gragert et al. (2013). An artificial SUPER-population aggregating all haplotype frequencies has been generated.

SUPPLEMENTARY FIGURE S2 Broad ancestry categories in the UNC dataset. The 9,471 analyzed samples from a dataset provided by University of North Carolina at Chapel Hill varied in their self-reported ancestry categories. Most samples had a self-identified CAU or AFA ancestry.

SUPPLEMENTARY FIGURE S3 Unphased HLA genotyping methods do not identify the maternal and paternal haplotypes but only provide a list of HLA genes. A high number of combined haplotypes can thus explain the provided typing result.

SUPPLEMENTARY FIGURE S4 For haplotypes not present in the frequency table, a stepwise removal of linkage between HLA loci is considered by the PIRCHE imputation algorithm.

SUPPLEMENTARY FIGURE S5 Imputation performance based on number of allele mismatch. Heatmaps indicating different evaluation metrics for comparing the imputed genotypes with the ground-truth genotypes. Both the results for the patients and donors were used, considering that only the actual performance of the imputation is evaluated here. The results were evaluated with the AUC score **(A)**, Brier score **(B)**, weighted F1-score **(C)** and balanced accuracy **(D)**. The scaling differs between the different evaluation metrics: in panels **(A, C, D)** a higher score indicates better performance, but a Brier score **(B)** measures mean-squared error, and thus lower is better. In all panels a darker blue color indicates better observed performance.

SUPPLEMENTARY FIGURE S6 Imputation Quality in Strict Imputation. Heatmap **(A)** shows relative rates of failed imputation, where valid haplotype pairs were not found based on ancestry assumptions (lower is better). Rates of failure are higher than in non-strict imputation (Figure 2A). Heatmap **(B)** shows mean allele-level mismatches between the high-resolution genotype and the successfully single imputed genotype (lower is better). In strict imputation we observed fewer allele mismatches in general in the (comparatively rare) successful imputations, compared with non-strict imputation.

SUPPLEMENTARY FIGURE S7 Comparison of proportions of noticeable PIRCHE-II deviations between the aggregated multiple imputed context and the single imputed context. Panels **(A, B)** show deviations in donor imputation, while panels **(D, E)** show deviations in patient imputation. Panels **(C, F)** indicate a comparison between deviations between the aggregated and single imputed contexts.

SUPPLEMENTARY FIGURE S8 Comparison of proportions of noticeable PIRCHE-II deviations between the aggregated multiple imputed context and the single imputed context in strict imputation. Panels **(A, B)** show deviations in donor imputation, while panels **(D, E)** show deviations in patient imputation. Panels **(C, F)** indicate a comparison between deviations between the aggregated and single imputed contexts.

SUPPLEMENTARY FIGURE S9 Comparison of proportions of noticeable Snow deviations between the aggregated multiple imputed context and the single imputed context. Panels **(A, B)** show deviations in donor imputation, while panels **(D, E)** show deviations in patient imputation. Panels **(C, F)** indicate a comparison between deviations between the aggregated and single imputed contexts.

SUPPLEMENTARY FIGURE S10 Comparison of proportions of noticeable Snow deviations between the aggregated multiple imputed context and the single imputed context in strict imputation. Panels **(A, B)** show deviations in donor imputation, while panels **(D, E)** show deviations in patient imputation. Panels **(C, F)** indicate a comparison between deviations between the aggregated and single imputed contexts.

SUPPLEMENTARY FIGURE S11 Imputing pairs of low-resolution HLA data. This figure shows a pessimistic estimation of risk, by imputing the patient and donor from low-resolution genotypes. Lines are separated by whether or not the patient and donor used a correct ancestry assumption in imputation. We observe in all cases that risk is reduced when the correct ancestry is assumed for both the patient and donor, compared to when both are imputed under wrong ancestry assumptions.
