# Supplementary material for: Quantifying uncertainty of molecular mismatch introduced by mislabeled ancestry using haplotype-based HLA genotype imputation
Source: Front Genet. 2024 Sep 25;15:1444554. doi: 10.3389/fgene.2024.1444554 (PMC11461215; doi:10.3389/fgene.2024.1444554)
Supplement: Supplementary file 1 [file DataSheet1.pdf]

Supplementary Materials

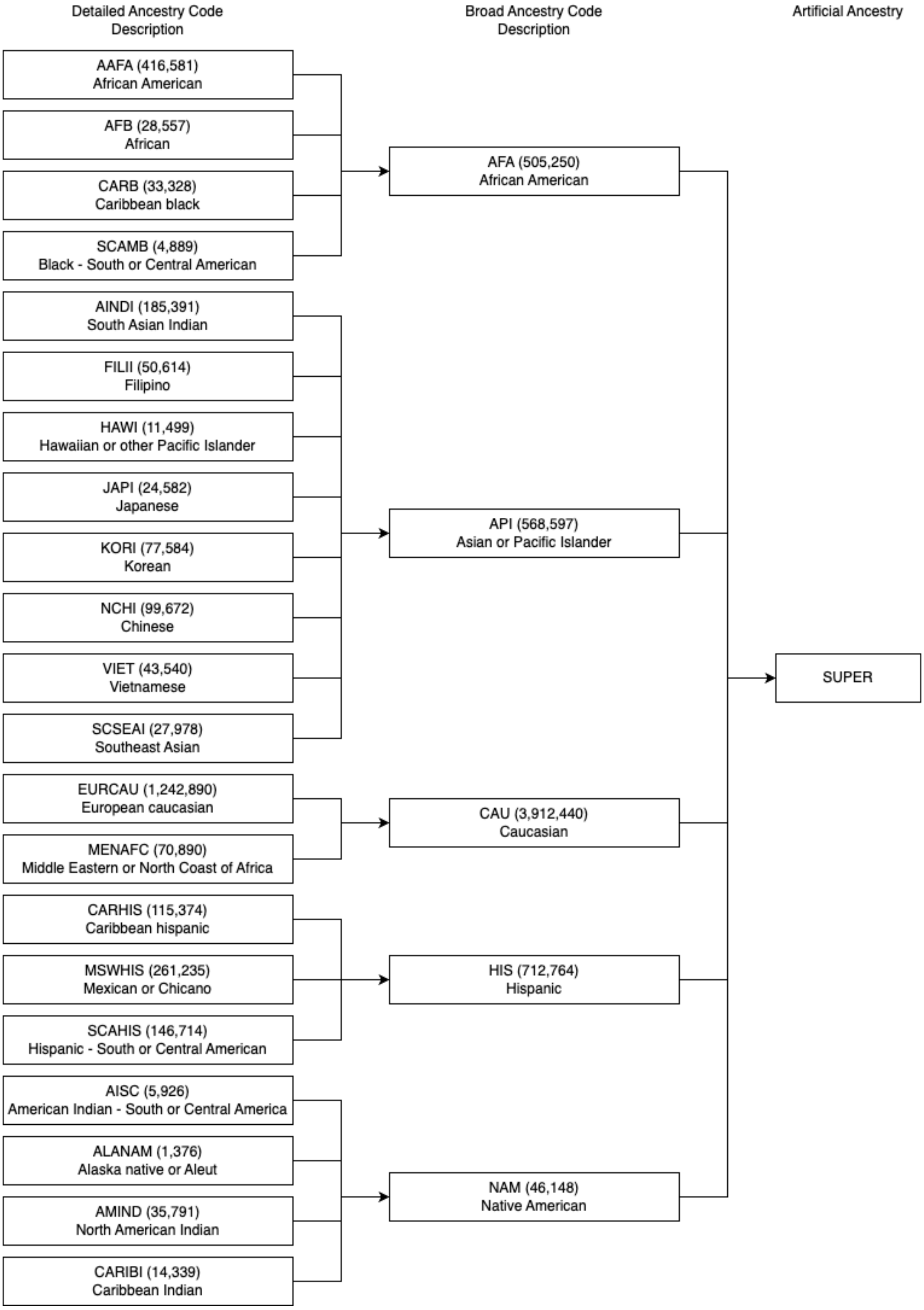

Supplementary Figure 1: Twenty-one detailed ancestry categories and descriptions and five broad ancestry categories were considered as suggested by (Gragert et al., 2013). In parentheses is the number of individuals from which these haplotype frequencies were generated, as shown in tables 1 and 2 of Gragert *et al*. An artificial SUPER-population aggregating all haplotype frequencies has been generated.

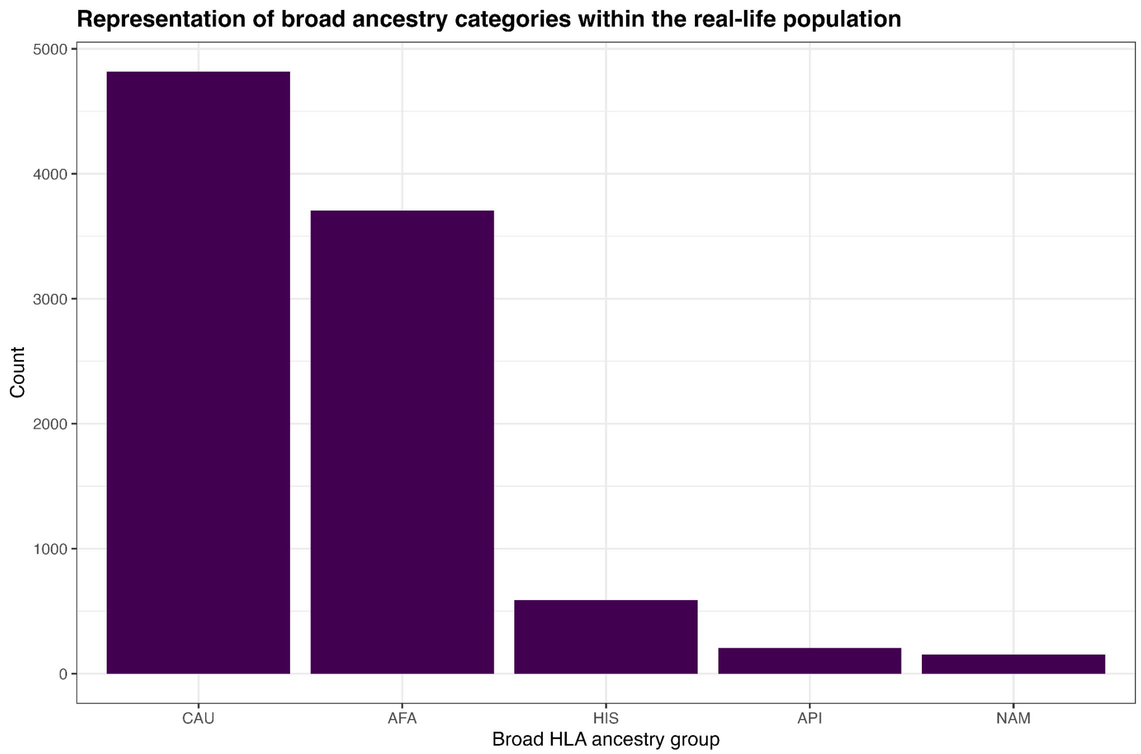

Supplementary Figure 2: Broad ancestry categories in the UNC dataset
The 9471 analyzed samples from a dataset provided by University of North Carolina at Chapel Hill varied in their self-reported ancestry categories. Most samples had a self-identified CAU or AFA ancestry.

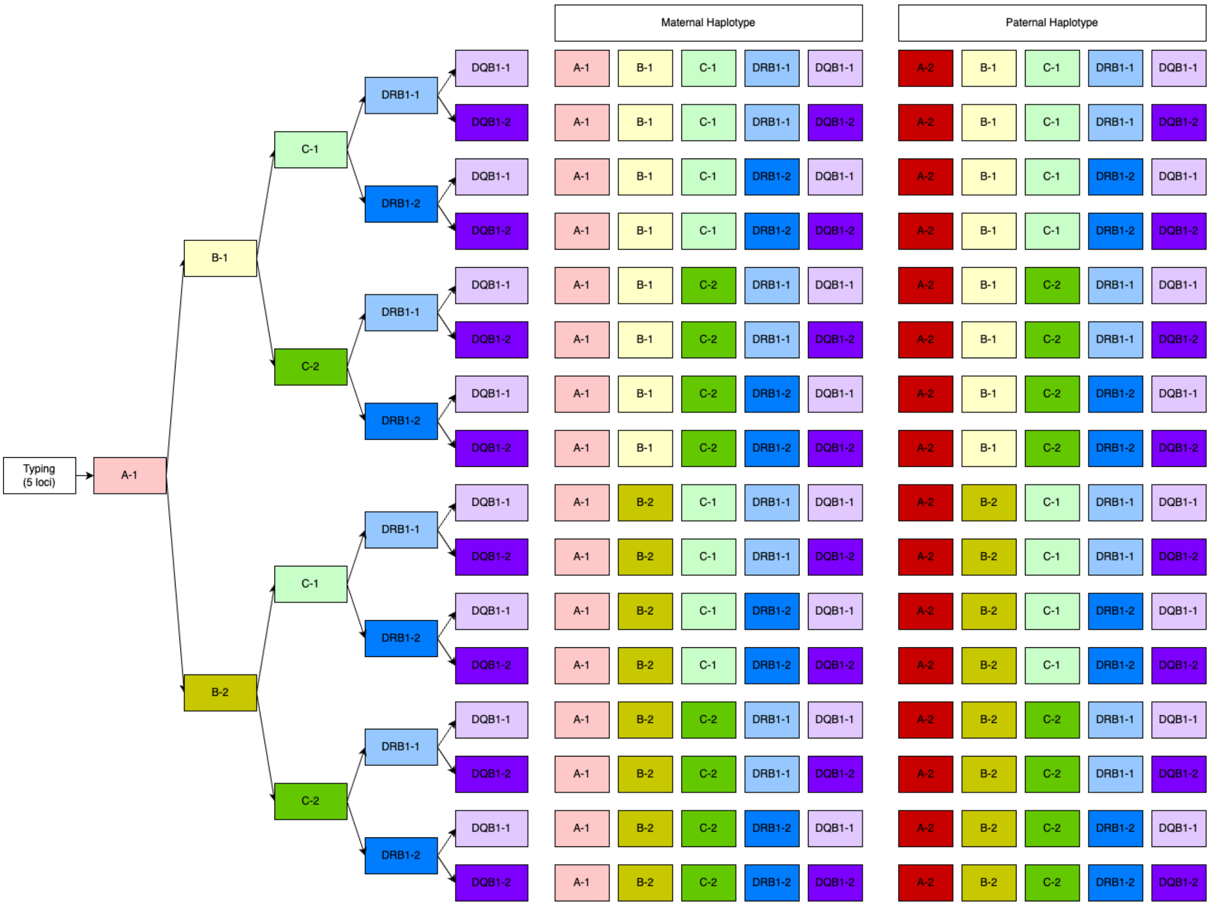

Supplementary Figure 3: Unphased HLA genotyping methods do not identify the maternal and paternal haplotypes but only provide a list of HLA genes. A high number of combined haplotypes can thus explain the provided typing result.

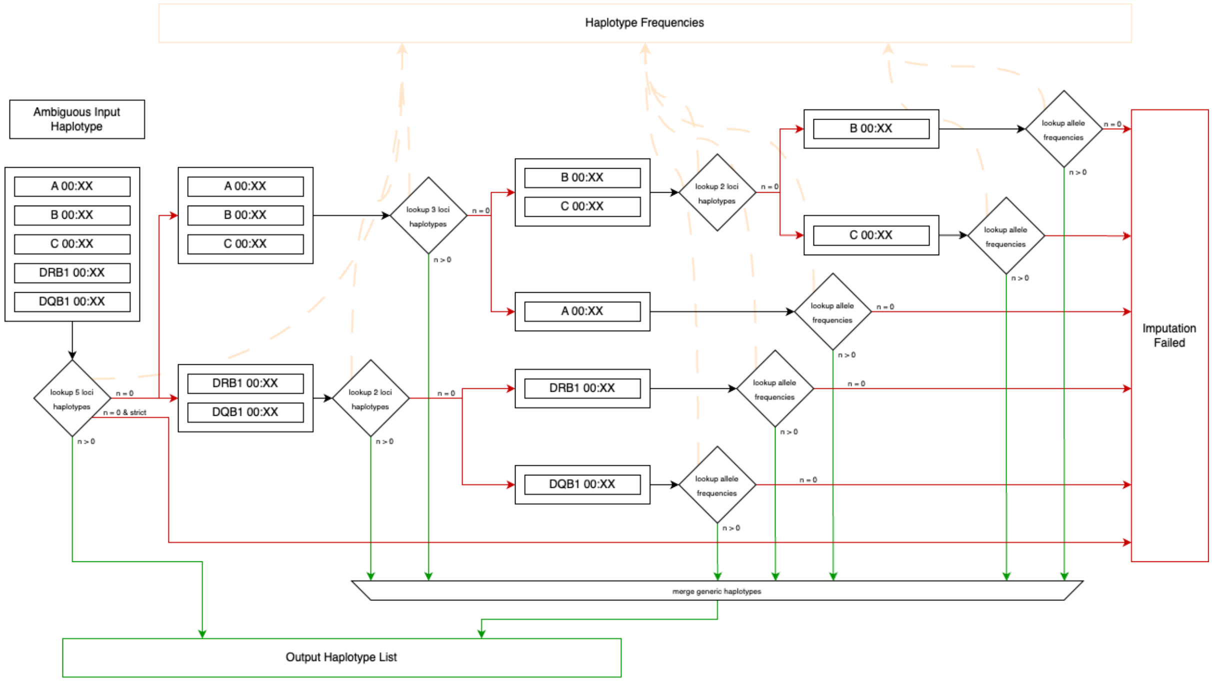

Supplementary Figure 4: For haplotypes not present in the frequency table, a stepwise removal of linkage between HLA loci is considered by the PIRCHE imputation algorithm.

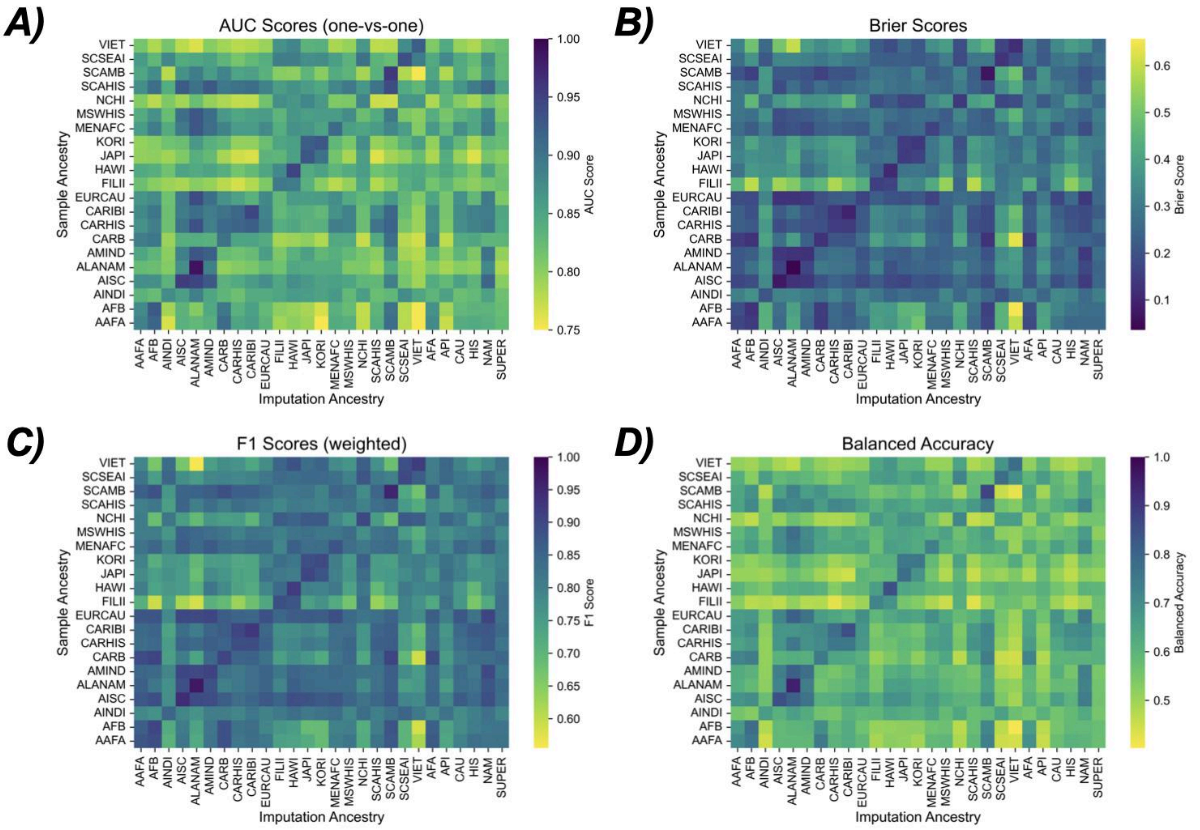

Supplementary Figure 5: Imputation performance based on number of allele mismatch.

Heatmaps indicating different evaluation metrics for comparing the imputed genotypes with the ground-truth genotypes. Both the results for the patients and donors were used, considering that only the actual performance of the imputation is evaluated here. The results were evaluated with the AUC score (A), Brier score (B), weighted F1-score (C) and balanced accuracy (D). The scaling differs between the different evaluation metrics: in panels (A,C, and D) a higher score indicates better performance, but a Brier score (B) measures mean-squared error, and thus lower is better. In all panels a darker blue color indicates better observed performance.

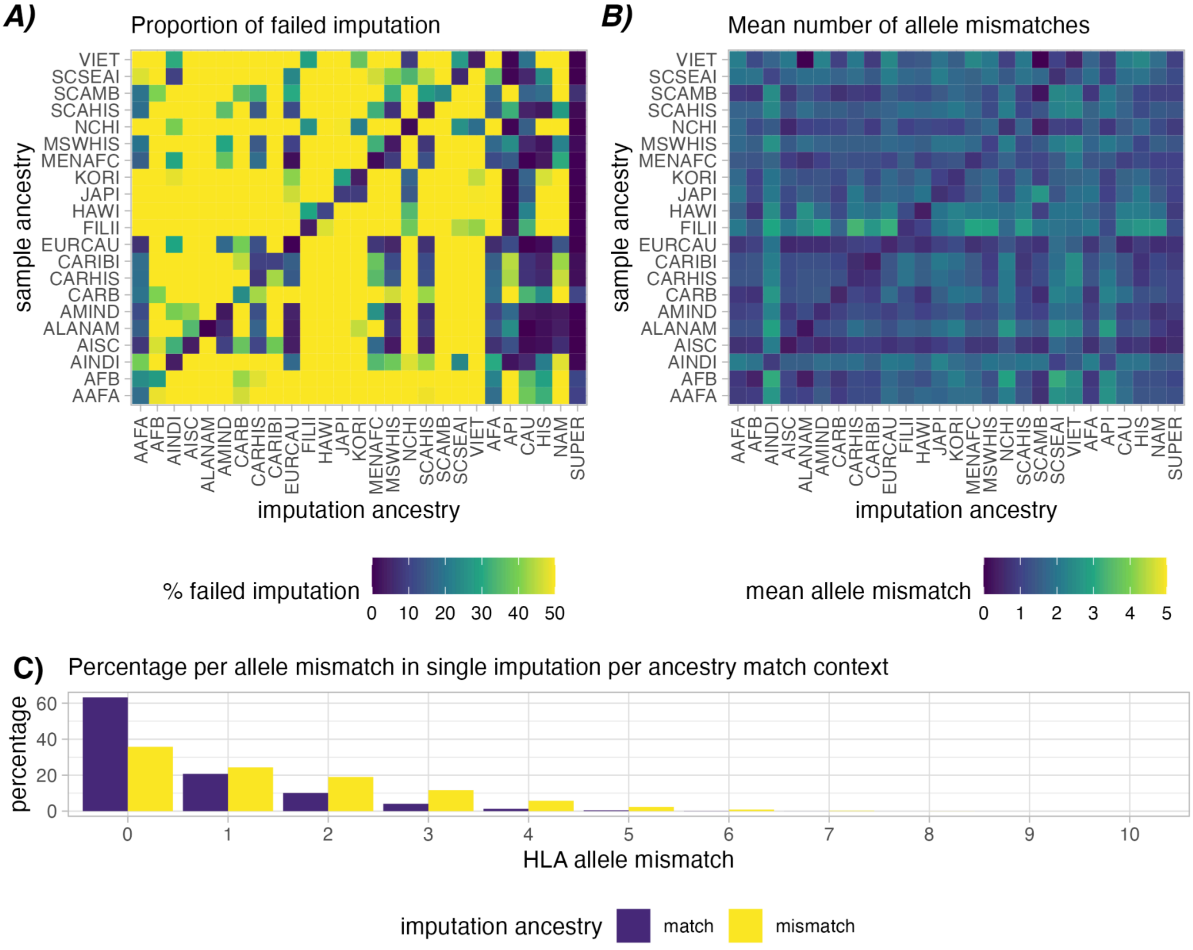

Supplementary Figure 6: Imputation Quality in Strict Imputation.
Heatmap A shows relative rates of failed imputation, where valid haplotype pairs were not found based on ancestry assumptions (lower is better). Rates of failure are higher than in non-strict imputation (Figure 2A). Heatmap B shows mean allele-level mismatches between the high-resolution genotype and the successfully single imputed genotype (lower is better). In strict imputation we observed fewer allele mismatches in general in the (comparatively rare) successful imputations, compared with non-strict imputation.

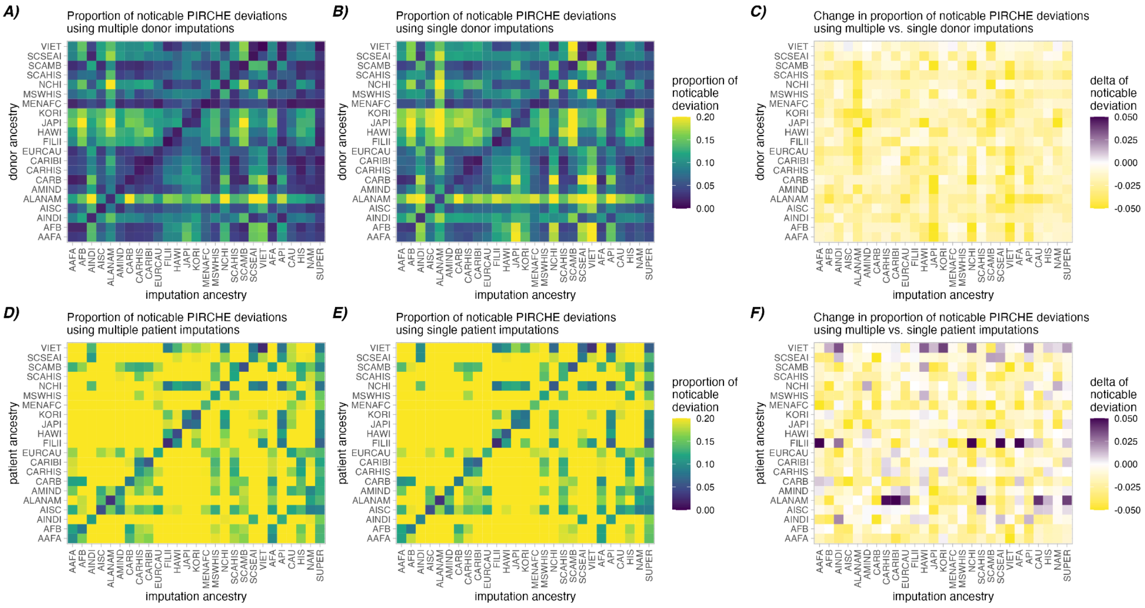

Supplementary Figure 7: Comparison of proportions of noticeable PIRCHE-II deviations between the aggregated multiple imputed context and the single imputed context. Panels A) and B) show deviations in donor imputation, while panels D) and E) show deviations in patient imputation. Panels C) and F) indicate a comparison between deviations between the aggregated and single imputed contexts

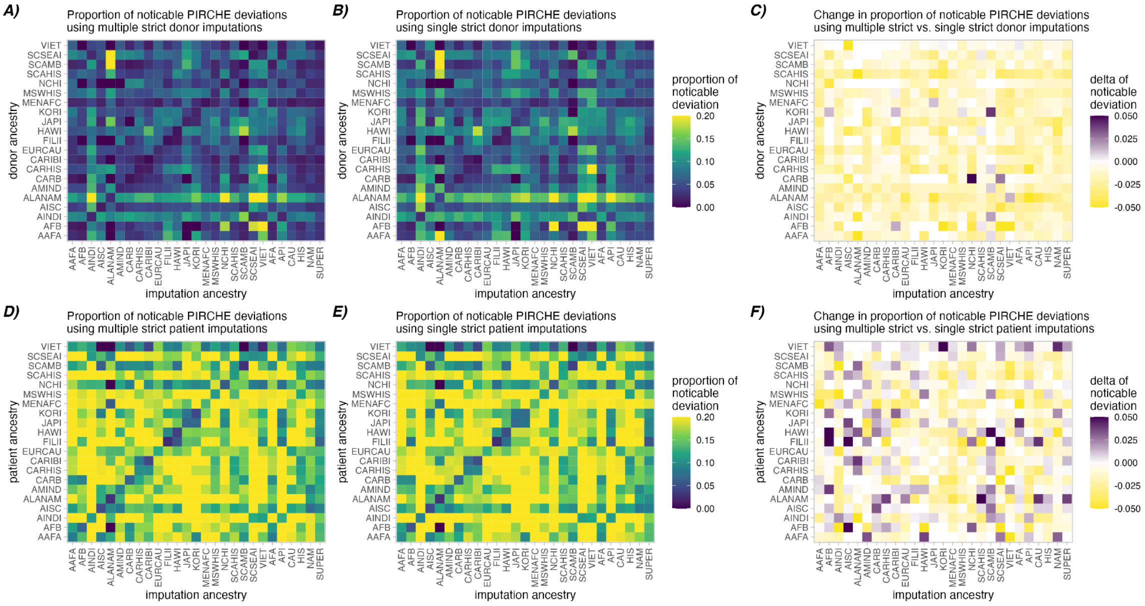

Supplementary Figure 8: Comparison of proportions of noticeable PIRCHE-II deviations between the aggregated multiple imputed context and the single imputed context in strict imputation. Panels A) and B) show deviations in donor imputation, while panels D) and E) show deviations in patient imputation. Panels C) and F) indicate a comparison between deviations between the aggregated and single imputed contexts

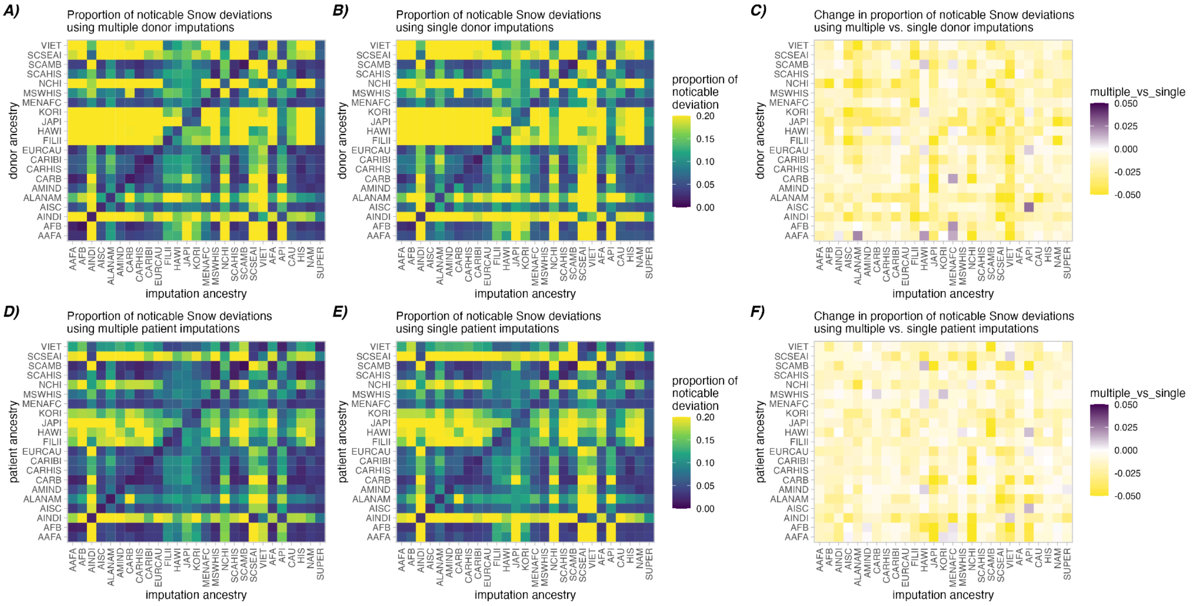

Supplementary Figure 9: Comparison of proportions of noticeable Snow deviations between the aggregated multiple imputed context and the single imputed context. Panels A) and B) show deviations in donor imputation, while panels D) and E) show deviations in patient imputation. Panels C) and F) indicate a comparison between deviations between the aggregated and single imputed contexts

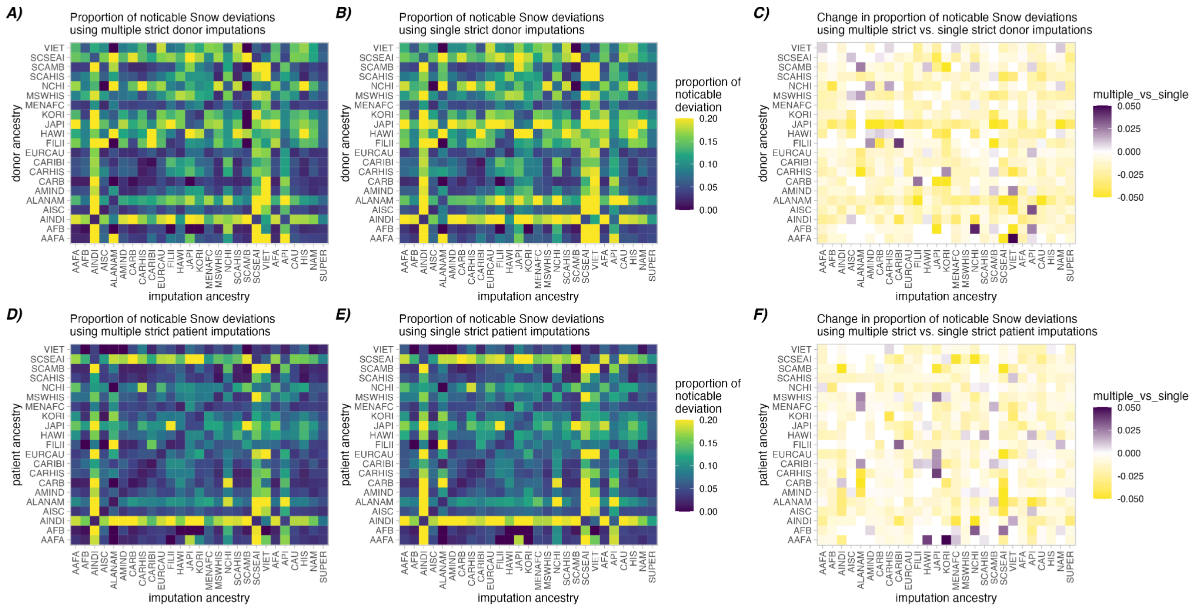

Supplementary Figure 10: Comparison of proportions of noticeable Snow deviations between the aggregated multiple imputed context and the single imputed context in strict imputation. Panels A) and B) show deviations in donor imputation, while panels D) and E) show deviations in patient imputation. Panels C) and F) indicate a comparison between deviations between the aggregated and single imputed contexts

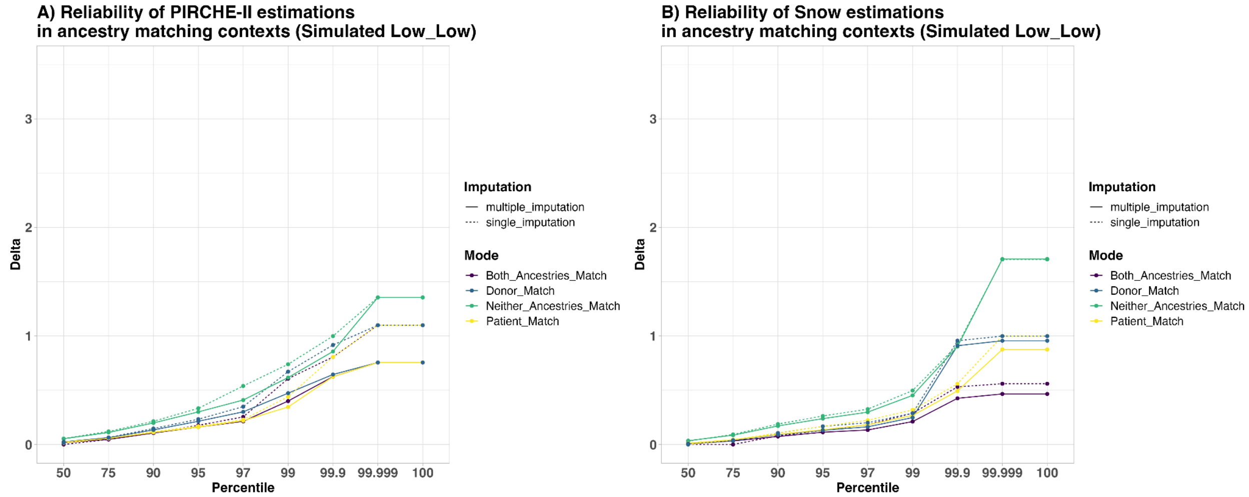

Supplementary Figure 11: Imputing pairs of low-resolution HLA data

This figure shows a pessimistic estimation of risk, by imputing the patient and donor from low-resolution genotypes. Lines are separated by whether or not the patient & donor used a correct ancestry assumption in imputation. We observe in all cases that risk is reduced when the correct ancestry is assumed for both the patient and donor, compared to when both are imputed under wrong ancestry assumptions.
